# Epidemiology of Latent Tuberculosis in Moroccan Healthcare Workers: Prevalence Patterns and Predisposing Factors

**DOI:** 10.7759/cureus.75740

**Published:** 2024-12-15

**Authors:** Abdelhalim Boucaid, Adil Zegmout, Mohamed Bhairis, Mouaad Amraoui, Azzeddine Laaraje, El Hassane Kabiri, Ismail Rhorfi

**Affiliations:** 1 Pulmonology, Mohammed V Military Training Hospital, Rabat, MAR; 2 Thoracic Surgery, Mohammed V Military Training Hospital, Rabat, MAR; 3 Pediatrics, Mohammed V Military Training Hospital, Rabat, MAR

**Keywords:** healthcare workers, latent tuberculosis infection, morocco, occupational health, quantiferon-tb gold, risk factors

## Abstract

Background: Among occupational hazards in healthcare settings, latent tuberculosis infection (LTBI) ranks as a major concern, particularly threatening healthcare workers (HCWs) in nations grappling with intermediate to high tuberculosis (TB) rates. Our study was conducted in Morocco, a country characterized by widespread Bacillus Calmette-Guérin (BCG) vaccination and a moderate TB burden of 93 cases per 100,000 inhabitants in 2022. We examined both the prevalence of LTBI among Moroccan HCWs and its various risk factors.

Methods: A cross-sectional study was conducted from August 2022 to October 2024 in two Moroccan hospitals. One hundred forty-seven HCWs were recruited and screened for LTBI using the QuantiFERON-TB Gold In-Tube (QFT-GIT) test. Data on demographics, occupational characteristics, and potential risk factors were collected through standardized questionnaires. Statistical analysis included univariate and multivariate logistic regression to identify factors associated with LTBI.

Results: The overall prevalence of LTBI was 32.65% (48/147). Multivariate analysis identified several independent risk factors: male gender (OR: 2.84; 95% CI: 1.54-5.22; p<0.001), age above 50 years (OR: 4.58; 95% CI: 1.50-13.90; p=0.007), smoking status (OR: 4.07; 95% CI: 1.63-10.14; p=0.003), and family history of TB (OR: 4.71; 95% CI: 1.33-16.65; p=0.016). Notably, neither specific work areas nor job categories were identified as significant risk factors in the final multivariate model.

Conclusions: The observed LTBI prevalence among HCWs in Morocco demonstrates concordance with epidemiological data from comparable intermediate-burden nations. The elucidation of predisposing factors, with particular emphasis on non-occupational determinants, underscores the imperative for implementing systematic surveillance protocols and World Health Organization (WHO)-sanctioned infection prevention measures within healthcare facilities. These epidemiological findings provide an empirical foundation for the formulation and optimization of TB control strategies specifically tailored to the occupational health needs of Morocco's healthcare workforce.

## Introduction

In 2022, tuberculosis (TB), although preventable and typically curable, ranked second among infectious diseases in global mortality, exceeded only by COVID-19, and claimed approximately twice as many lives as HIV/AIDS.

The annual global incidence of TB exceeds 10 million cases. Transmission occurs via airborne droplets when individuals with active TB release bacterial particles, particularly through respiratory actions such as coughing. While the primary site of infection is pulmonary, TB can manifest in various extrapulmonary locations.

Current epidemiological estimations suggest that between 5% and 10% of infected individuals will develop active TB during their lifetime [1].

Latent tuberculosis infection (LTBI) occurs when a person is infected with TB bacteria but shows no symptoms and cannot spread the disease.

According to mathematical modeling research published in 2016 [2], approximately one-fourth of the global population harbors LTBI, constituting a substantial reservoir for potential disease activation [2].

Recent studies are challenging traditional estimates about how common LTBI is, suggesting the numbers may be lower than previously thought. This new perspective is supported by two main findings: The human body can naturally clear the infection on its own without treatment, and current testing methods (tuberculin skin test (TST) and interferon-gamma release assays (IGRAs)) have a significant limitation, that is, they cannot tell the difference between someone who currently has an inactive infection and someone who had an infection in the past but has cleared it. Because of these factors, especially the testing limitation, researchers believe we may be overestimating how many people actually have LTBI [1,3,4].

Among the people at risk, healthcare workers (HCWs) are at the top of the list, due to exposure to diagnosed and undiagnosed TB patients. This risk is proportionally more alarming in low- and middle-income countries because of both increased exposure and lack of preventive measures, such as poor workplace ventilation and inadequate precautions [5]. Therefore, the screening of this population (HCWs) for LTBI and active TB disease is critical in infection control programs in hospitals [6].

The initial diagnostic tool developed for LTBI detection was the in vivo TST. This method demonstrates high sensitivity (77%; 95% CI: 71-82) but exhibits limited specificity (59%; 95% CI: 46-73) [7], attributed partially to its cross-reactive nature with Bacillus Calmette-Guérin (BCG) vaccination and non-tuberculous mycobacterial (NTM) infections [8].

In response to these diagnostic limitations, the development of IGRAs has provided enhanced specificity compared to TST through in vitro testing. This improved specificity is achieved through the utilization of antigens distinct from those present in BCG vaccine strains and common NTM species [9].

The 2022 surveillance data from Morocco's National TB Program revealed 35,000 diagnosed cases of TB encompassing all forms. Notwithstanding the country's achievement of an 87% treatment success rate, World Health Organization (WHO) estimations indicate that annual TB-related mortality in Morocco persists at 2,800 cases [10].

We investigated the prevalence and risk factors for LTBI assessed by IGRAs in HCWs from Morocco, a low-middle-income country with an intermediate annual incidence of TB (93/100,000 in 2022) and high BCG vaccination coverage.

## Materials and methods

Study design and population

A cross-sectional study was conducted from August 2022 to October 2024 in two Moroccan cities, Laayoune and Dakhla. HCWs were recruited from two hospitals, the Military Hospital Mohammed VI of Dakhla and the Military Hospital Hassan II of Laayoune.

During the recruitment period, the incidence of TB remained stable at around 90 per 10,000 inhabitants. The inclusion criteria for the study were age between 20 and 65 and agreement to participate. The exclusion criteria for the study were a personal history of TB, pregnancy, known neoplastic disease or renal failure, HIV infection, known viral hepatitis B or C, and immuno-suppressive treatment.

Using a questionnaire (see Appendices), various parameters were collected for each candidate, including age and sex, co-morbidities (asthma, diabetes, etc.), smoking habits, familial history of TB and personal history of TB infection or disease, BCG vaccination status, and workplace. Furthermore, the workplace was subdivided into four categories: non-clinical (laboratory, administration, etc.) and surgical departments (low risk of transmission), clinical departments (medium risk of transmission), and pulmonology departments (high risk of transmission). The function within the hospital was subdivided into three categories: medical staff, paramedical staff (nurses and laboratory technicians), and non-medical staff (secretary and administrator).

All participating HCWs reported receiving BCG immunization in the neonatal period. The study population comprised HCWs who satisfied the inclusion parameters and submitted completed questionnaire data.

Ethics statement

This study was conducted in accordance with the ethical principles of the Declaration of Helsinki. The study protocol was reviewed and approved by the Local Ethics Committee of the Sanitary Delegation of Oued Eddahab. The study was also registered in the Research Registry (Research Registry UIN: researchregistry10864). Written informed consent was obtained from all participants after being informed about the study objectives, procedures, potential risks and benefits, and their right to withdraw at any time without consequences.

All data were collected anonymously and handled confidentially. Personal identifiers were removed from the dataset before analysis to ensure participant privacy. Participants who tested positive for LTBI were provided appropriate counseling and referred for further medical evaluation and preventive therapy according to national guidelines. The study procedures adhered to institutional biosafety protocols, and all testing was performed by trained personnel following standard precautions.

Access to the collected data was restricted to the research team members. The results are presented in aggregate form to prevent the identification of individual participants. No monetary or other compensation was provided to participants. This research was conducted as part of routine occupational health screening, causing minimal additional burden to participants.

QuantiFERON-TB Gold In-Tube (QFT-GIT) (QIAGEN, Venlo, the Netherlands) test assay

In our study, the QFT-GIT technique was used, and a blood sample was taken by venipuncture from all candidates in order to fill the three specific tubes (negative control/TB antigen designed to stimulate lymphocytes/positive control with mitogen).

Three results are possible: positive, i.e., ≥0.35 IU/ml, negative, i.e., <0.35 IU/ml, and indeterminate.

Statistical analysis

Statistical analysis was performed using jamovi (Version 2.3) [Computer Software] (retrieved from https://www.jamovi.org); descriptive statistics included mean and standard deviation for quantitative variables and frequency and percentage for qualitative variables. Analytical statistics involved the chi-squared test or Fisher's exact test for qualitative variables and Student's t-test for quantitative variables. Correlation, univariate, and multivariate logistic regression analyses were conducted, with p<0.05 considered statistically significant.

## Results

Following the agreement of the candidates, a total of 147 health personnel working at the Military Hospital Mohammed VI of Dakhla and the Military Hospital Hassan II of Laayoune were selected.

The interview revealed no functional or general signs, and the candidates underwent a thorough clinical examination and a chest X-ray. No cases of active TB were diagnosed, and no cases of active TB were detected during the study period.

The age of our candidates ranged from 20 to 62, with an average age of 34±5.2. Twenty-one (14.28%) candidates were aged between 20 and 35 years, 81 (55.1%) were aged between 35 and 50 years, and 45 (30.61%) were aged over 50 years. Men are in the majority, with 75 (51.03%) candidates, and the sex ratio was 1.04. Nineteen (12.9%) candidates had co-morbidities, and six (4.08%) of them had diabetes. Smoking was found in 17 (11.56%) candidates, compared with 130 (88.44%) nonsmoking candidates, a recent familial history of TB infection was found in 14 (9.52%) candidates, and all candidates had been vaccinated as children with BCG.

In terms of the workplace, 55 (37.41%) candidates were working in non-clinical departments, 31 (21.08%) in surgical departments, 40 (27.21%) in clinical departments, and 21 (14.28%) in pulmonary departments.

Concerning the job category, 36 (24.5%) candidates were doctors, paramedical staff were 69 (47%), and non-medical staff were 42 (28.5%).

QFT was negative in 98 (66.6%) and positive in 48 (32.65%), giving a prevalence of positivity of 32.65%, and the result was indeterminate in one participant (Table 1).

**Table 1:** Baseline characteristics of 147 healthcare workers BCG: Bacillus Calmette-Guérin; TB: tuberculosis; QFT-GIT: QuantiFERON-TB Gold In-Tube

Characteristics	n (%)
Gender	
Male	75 (51.03)
Female	72 (48.97)
Age groups (years)	
20-35	21 (14.28)
35-50	81 (55.10)
50-65	45 (30.61)
BCG vaccination status	
No	0 (0)
Yes	147 (100)
Co-morbidities	
No	128 (87.1)
Yes	19 (12.9)
Smoking status	
No	130 (88.44)
Yes	17 (11.56)
Family history of TB	
No	133 (90.48)
Yes	14 (9.52)
Work area	
Non-clinical department	55 (37.4)
Surgical department	31 (21.08)
Clinical department	40 (27.21)
Pulmonology department	21 (14.28)
Occupation	
Non-medical staff	42 (28.57)
Paramedical staff	69 (46.93)
Medical staff	36 (24.5)
QFT-GIT	
Negative	98 (66.6)
Positive	48 (32.65)

The univariable analysis identified significant relationships between positive QFT-TB status and male gender (OR=3.13; 95% CI: 1.90-5.13; p<0.001), age above 50 years (OR=5.70; 95% CI: 2.21-14.68; p<0.001), family history of TB (OR=4.07; 95% CI: 1.33-12.48; p=0.014), smoking status (OR=4.32; 95% CI: 2.08-8.99; p<0.001), being a doctor (OR=0.37; 95% CI: 0.19-0.73; p=0.04), and also working in specific department like clinical department (OR=0.45; 95% CI: 0.24-0.83; p=0.011).

In multivariable analysis, all the variables remained significant except the job category as doctors (OR=0.48; 95% CI: 0.18-1.25; p=0.136) and the work in the clinical department (OR=0.83; 95% CI: 0.35-1.92; p=0.667) (Table 2).

**Table 2:** Risk factors associated with a positive QFT-GIT result in 147 healthcare workers TB: tuberculosis; CI: confidence interval; QFT-GIT: QuantiFERON-TB Gold In-Tube; OR uni: odds ratio obtained in a univariable regression; p uni: p-value corresponding to the coefficient obtained in a univariable regression; OR mult: odds ratio obtained in a multivariable regression including all the variables explored in univariable analyses; p mult: p-value corresponding to the coefficient obtained in a multivariable regression including all the variables explored in univariable analyses

Characteristics	Total n-1=146	QFT(+)	QFT(-)	OR uni (95% CI)	P uni	OR mult (95% CI)	P mult
Gender							
Female	72	15	57	Ref		Ref	
Male	74	32	42	3.13 (1.90-5.13)		2.84 (1.54-5.22)	
Age groups (years)							
20-35	20	4	16	Ref		Ref	
35-50	81	22	59	2.32 (0.92-5.85)	0.073	2.131 (0.75-6.05)	0.155
50-65	45	22	23	5.70 (2.21-14.68)		4.582 (1.50-13.90)	0.007
Co-morbidities							
No	127	42	85	Ref		Ref	
Yes	19	5	14	0.6 (0.27-1.31)	0.202	0.457 (0.14-1.44)	0.184
Smoking status							
No	132	40	92	Ref		Ref	
Yes	14	9	5	4.32 (2.08-8.99)		4.07 (1.63-10.14)	0.003
Family history of TB							
No	139	42	97	Ref		Ref	
Yes	7	5	2	4.07 (1.33-12.48)	0.014	4.71 (1.33-16.65)	0.016
Diabetes							
No	140	46	94	Ref		Ref	
Yes	6	2	4	0.94 (0.28-3.13)	0.923	1.374 (0.24-7.62)	0.717
Work area							
Non-clinical department	55	22	33	Ref		Ref	
Surgical department	31	7	24	0.37 (0.18-0.74)	0.07	0.27 (0.104-0.70)	0.12
Clinical department	39	11	28	0.45 (0.24-0.83)	0.011	0.83 (0.35-1.92)	0.667
Pulmonology department	21	7	14	0.75 (0.38-1.51)	0.435	1.84 (0.69-4.85)	0.218
Occupation							
Non-medical staff	41	19	22	Ref		Ref	
Paramedical staff	69	21	48	0.58 (0.33-1.006)	0.053	0.92 (0.42-2.01)	0.835
Medical staff	36	9	27	0.379 (0.19-0.73)	0.004	0.48 (0.18-1.25)	0.136

## Discussion

This research explores the prevalence of LTBI in the healthcare workforce of Morocco, a lower-middle-income country distinguished by intermediate TB endemicity and universal BCG vaccination implementation.

This study focused on HCWs living in the kingdom's two southernmost administrative regions. The global prevalence of LTBI was 24.8% (95% CI: 19.7-30.0) and 21.2% (95% CI: 17.9-24.4) based on IGRA and a 10-mm TST cut-off, respectively. The prevalence estimates correlated well with WHO incidence rates [11].

The estimated prevalence of LTBI in the general Moroccan population ranges from 25% to 45%, varying significantly by region, demographic factors, and the test used. Urban areas demonstrate higher prevalence rates (35-45%) compared to rural regions (20-30%) [12]. In our study of this high-risk population, the prevalence of LTBI was 32.65%.

This value is consistent with an anterior study done in Morocco where the prevalence was 40.7% [13] and contrasts with prevalences observed among HCWs living in other medium TB incidence countries such as Iran at 16.92% [14], Colombia 62.1% [15], Mexico 70% [16], and Malaysia 61.7% [17]. In a meta-analysis from Brazil, the prevalence of LTBI among primary HCWs was reported to be around 27% [18].

Male gender is a well-known risk factor for active TB disease due at least in part to socio-cultural causes as well as biological differences between sexes. In our study, the male gender was associated with a high risk of LTBI (OR=2.84; 95% CI: 1.54-5.22; p<0.001); this was also the case in different studies in multiple medium-burden countries [14,19].

Multiple potential explanations for male predominance can be proposed: (1) behavioral factors (lower compliance with personal protective equipment, higher risk-taking behavior), (2) occupational factors (different roles in patient care, different job assignments), and (3) biological factors (potential immunological differences, hormonal influences, genetic susceptibility). However, it's important to note that while these factors are discussed in the literature, their relative contributions may vary across different populations and settings. The exact mechanisms behind gender differences in TB susceptibility continue to be an area of active research [20,21].

Most studies demonstrate an age effect, increasing LTBI risk with advancing age. In our case, all the HCWs were engaged from the age of 18 and worked in those health institutions until the time of the study, the age was correlated to the exposure duration, the age group above 50 years was associated with a high risk of LTBI (OR=4.58; 95% CI: 1.50-13.90; p=0.007) compared to other age groups, and those results align with other studies even if the cut-offs were different [16,18]. Multiple explanatory factors can be proposed: (1) cumulative exposure, (2) historical factors (changes in infection control measures, the evolution of preventive protocols), and (3) biological factors (immunological changes with aging, increased co-morbidities, and altered host response) [22,23].

Smoking was identified in our study as a risk factor for LTBI (OR=4.07; 95% CI: 1.63-10.14; p=0.003), and this aligns with the literature. Lindsay et al. showed that current smokers have a higher risk of LTBI (OR=2.31; 95% CI: 1.58-3.38) than nonsmokers (reference group) and even passive smoking exposure increases the risk (OR=1.53; 95% CI: 1.07-2.18) [24].

Other studies showed a dose-response relationship with pack/years [25] and recognized a temporal dose-response pattern [26]. Multiple factors were described in the literature to explain this relation, and those factors can be divided into direct biological effects of smoking (impaired mucociliary clearance, compromised alveolar macrophage function, altered immune response, and chronic inflammation) and indirect effects of smoking (modified exposure behaviors and associated lifestyle factors) [27].

In medium-burden countries, a family history of TB was always associated with a high risk of LTBI, like in Brazil (OR = 2.03; 95% CI: 1.36-3.03), and our study also highlighted this association (OR=4.71; 95% CI: 1.3-16.6; p=0.016) [19]. Several influencing factors were identified in the literature: (1) temporal factors (recent exposure vs old exposure), (2) exposure intensity (shared bedroom: highest risk, same household: moderate risk, and occasional contact: lower risk), (3) living conditions (ventilation, duration of cohabitation, bedroom sharing, and house size), and (4) index case characteristics (smear status, disease severity, treatment compliance, and drug resistance status) [28].

Statistical analysis failed to identify significant associations between LTBI status and occupational parameters, including both workplace setting and job categories, and this aligns with Molina-Gamboa et al.'s study in Mexico [15] and de Souza et al.'s study in Brazil [19]. We can suggest that non-occupational risks (such as age, gender, and smoking) may play a more important role than hospital-specific factors in the development of LTBI among HCWs in this cohort, and this also can be explained by the relatively small sample size. Conversely, several studies have documented heightened risk levels in specific occupational settings. TB laboratory personnel demonstrate a significantly elevated risk (OR=2.06; 95% CI: 1.35-3.17) [29], while HCWs with direct TB patient contact also show increased risk levels (OR=1.64; 95% CI: 0.74-3.64) [17]. Several factors intervene in this process like infection control measures: (1) administrative controls (presence vs absence of TB screening programs, patient isolation protocols, staff training programs), (2) environmental controls (ventilation systems, UV irradiation, negative pressure rooms), and (3) personal protective equipment (N95 mask availability and usage, compliance with protocols, training adequacy) [30]. 

In this cross-sectional study investigating LTBI among Moroccan HCWs, several methodological strengths merit attention, including the utilization of the highly specific QFT-GIT test and robust statistical analysis incorporating both univariate and multivariate approaches. The identification of non-occupational risk factors offers valuable insights into the existing literature on TB epidemiology in healthcare settings. However, several limitations warrant consideration. The relatively modest sample size (n=147) from only two hospitals may limit the generalizability of findings to the broader Moroccan healthcare workforce. The cross-sectional design precludes the establishment of temporal relationships between risk factors and LTBI development. Additionally, the absence of longitudinal follow-up data prevents the assessment of conversion rates and the dynamic nature of occupational exposure. The study's timing, extending into late 2024, encompasses potential variations in healthcare practices during the post-pandemic period, which might have influenced exposure patterns. 

Future research would benefit from larger, multi-center prospective studies incorporating detailed workplace exposure metrics and molecular epidemiological approaches to better delineate the occupational versus community-acquired transmission patterns in this population.

## Conclusions

This study revealed the prevalence of LTBI (32.65%). In this epidemiological and socioeconomic context, screening and searching for TB and LTBI among hospital HCWs is necessary for TB control efforts. The implementation of screening programs and infection control measures as recommended by the WHO can play an important role in identifying higher-risk cases and decreasing TB incidence among this decisive and important population.

## Appendices

Questionnaire for latent tuberculosis infection screening in healthcare workers

A. Demographic Information

1. Age: ____ years

2. Gender: □ Male □ Female

3. City/hospital: □ Military Hospital Mohammed VI of Dakhla □ Military Hospital Hassan II of Laayoune

B. Medical History

4. Bacillus Calmette-Guérin vaccination status: □ Yes □ No □ Unknown

    - If yes, age at vaccination: ____

5. Co-morbidities:

   □ None

   □ Diabetes

   □ Asthma

   □ Other (specify): ________

6. Smoking status:

   □ Never smoker

   □ Current smoker

   □ Former smoker

    - If current/former smoker: Number of cigarettes per day: ____

    - Duration of smoking: ____ years

C. Tuberculosis Exposure History

7. Family history of tuberculosis:

   □ Yes □ No

   - If yes, specify the relationship and when: ________

8. Known tuberculosis contact:

   □ Yes □ No

   - If yes, specify circumstances: ________

D. Occupational Information

9. Workplace category:

   □ Non-clinical departments (laboratory, administration)

   □ Surgical departments

   □ Clinical departments

   □ Pulmonology departments

10. Job category:

   □ Medical staff (doctor)

   □ Paramedical staff (nurse, laboratory technician)

   □ Non-medical staff (secretary, administrator)

11. Years of experience in healthcare: ____ years

E. Exclusion Criteria Verification

12. Do you have any of the following? (Check all that apply)

   □ Personal history of tuberculosis

   □ Pregnancy

   □ Known neoplastic disease

   □ Renal failure

   □ HIV infection

   □ Viral hepatitis B or C

   □ Current immunosuppressive treatment

F. Clinical Assessment (To Be Completed by a Healthcare Provider)

13. Physical examination results:

   - Chest X-ray findings: ________

   - Clinical signs: ________

14. QuantiFERON-TB Gold test results:

   □ Positive (≥0.35 IU/ml)

   □ Negative (<0.35 IU/ml)

   □ Indeterminate

Date of completion: ___/___/____

Signature of the healthcare provider: ________
